# Academic resilience in nursing students: a concept analysis

**DOI:** 10.1186/s12912-024-02133-2

**Published:** 2024-07-09

**Authors:** Yang Shen, Hanbo Feng, Xiaohan Li

**Affiliations:** School of Nursing, China Medical University, 77 Puhe Road, Shenbei New District, Shenyang, China

**Keywords:** Nursing students, Academic resilience, Concept analysis, Nursing education

## Abstract

**Background:**

Academic resilience is a crucial concept for nursing students to cope with academic challenges. Currently, there is significant variation in the description of the concept attributes of academic resilience among nursing students, which impedes the advancement of academic research. Therefore, it is essential to establish a clear definition of the concept of academic resilience for nursing students.

**Purpose:**

The purpose of this paper is to report the results of concept analysis of academic resilience of nursing students.

**Methods:**

The Rodgers evolutionary concept analysis was employed to test the attributes, antecedents, consequences and related concepts of academic resilience of nursing students. Walker and Avant’s method was utilized to construct a model case and provide empirical referents.

**Results:**

The findings indicate that the attributes of nursing students’ academic resilience include self-efficacy, self-regulation and recovery, and the antecedents include internal factors and external environmental factors. The consequences include adaptability, career maturity, adversity quotient level, probability of academic success, a sense of belonging to school and low levels of psychological distress.

**Conclusion:**

The systematic understanding of academic resilience among nursing students provides a pathway for nursing educators and students to enhance academic resilience, promote academic success, and establish a foundation for the training of more qualified nurses.

**Supplementary Information:**

The online version contains supplementary material available at 10.1186/s12912-024-02133-2.

## Introduction

Nursing is a scientific discipline that studies nursing theory and technology in the process of health promotion and disease prevention, with the aim of equipping students with knowledge, attitudes, and skills essential to the nursing profession [1]. Compared with students in other majors, nursing students often experience great pressure from unfamiliar clinical environments, the gap between professional theory and practice, unexpected emergencies, strained relationship with patients and their families, exposure to infectious diseases, as well as heavy workloads [2, 3]. The high levels of stress experienced by nursing students can negatively affect their overall well-being and academic performance [4–6], compromising their quality of life while increasing burnout rates [7]. Moreover, sustained pressure may impede critical thinking abilities, problem-solving skills, decision-making capabilities among nursing students thereby diminishing their motivation for learning and hindering academic achievement [4, 6, 8], ultimately failing to complete their professional learning. Students’ coping ability and academic success are closely related to their perception and handling of academic pressure. Therefore, it is particularly crucial to assist them in comprehending their academic pressure and difficulties and continuously enhancing nursing students’ psychological quality in order to cope with setbacks and achieve successful adaptation [9, 10].

Resilience refers to experiencing significant traumatic events and difficulties and still achieving positive developmental outcomes [11]. It is the process of adapting to major stressors and negative factors, as well as the strength to recover from stressful life events or successfully cope with negative experiences [12, 13]. There is evidence that resilience can effectively buffer the negative impact of individuals and contribute to improved individual happiness and life satisfaction [14–17]. It is essential for nursing students to possess a positive psychological quality in order to effectively cope with academic pressure [18, 19]. As resilience research continues to expand and evolve, its scope, focus, and application have diversified across different fields [10, 20].

Academic resilience is a relatively new concept. It is the manifestation of resilience in the field of education [3]. It can assist students in adapting to the demands of school and clinical settings, enabling them to overcome academic pressure [21]. Resilient students are able to maintain high academic achievement and perform well even when they are faced with the threat of failure or stressful situations [22]. Therefore, academic resilience is crucial for nursing students [23–25], and enhancing academic resilience may help students focus on their strengths and potentially reverse academic failure. Furthermore, it can also help more nursing students persist in their nursing education, thereby contributing much-needed nurses to the profession [3].

Academic resilience is a crucial concept for nursing students in managing academic challenges. However, there are significant differences in the depiction of the conceptual attributes of academic resilience among nursing students, which impedes the advancement of academic research [26, 27]. It is necessary to clarify the attributes and connotations of the concept in order to establish a foundation for planning academic resilience intervention measures for nursing students. This will ultimately improve the quality of nursing education and better meet the needs of students. Currently, there is no conceptual analysis of the academic resilience of nursing students. Therefore, the aim of this study is to explore the concept of academic resilience among nursing students.

## Methods

In this study, Rodgers [28] evolutionary concept analysis method was applied to explore and clarify the concept of academic resilience of nursing students. The main steps were to determine the concept evolution, application, definition, conceptual attributes, antecedents, consequences of nursing students’ academic resilience, distinguish related concepts of nursing students’ academic resilience. Walker and Avant’s [29] method was employed to construct a model case and provide empirical referents. The researchers initially read the articles included, focus on the context of the concept, surrogate and related terms, the attributes, antecedents, consequences, and examples, and then analyze the data collected.

### Search strategy

Search PubMed, Scopus, Embase, CINAHL, PsycINFO, Web of Science, ProQuest, Science Direct, CNKI, Wanfang Database, VIP database. Full search strategy can be found in the supplemental material.

### Selection criteria

Literature inclusion criteria: (1) The subjects were nursing students who were studying in schools or practicing in hospitals. (2) The research contents include the concept, defining characteristics, attributes, antecedents, influencing factors and consequences of academic resilience. Exclusion criteria: (1) The full text cannot be obtained. (1) Repeated reports. (2) Non-Chinese or non-English literature.

### Literature screening

The publications were imported into EndNote 20.0 software for de-duplication, the articles was selected independently by two researchers according to inclusion and exclusion criteria. If there was any disagreement, the two researchers would discuss it or invite a third researcher to join the discussion, and the included articles were ultimately determined.

## Results

### Search results

**Fig. 1:**
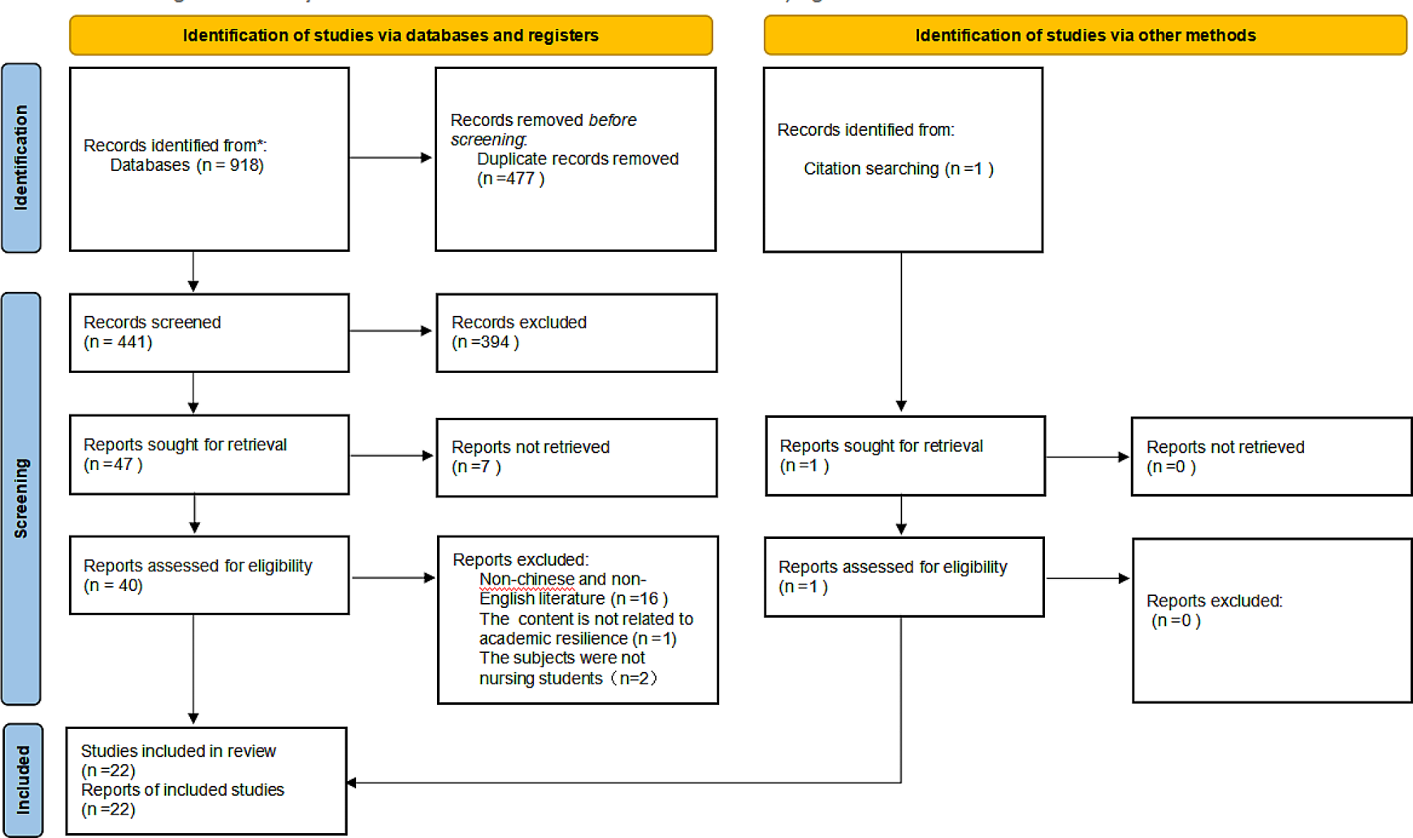
PRISMA diagram of literature search

A total of 918 literature were obtained. Among them, there are 741 English papers and 177 Chinese papers. After screening and exclusion according to the selection criteria, 22 literature were finally included. See Fig. 1 for PRISMA Diagram.

The 22 articles included in this study were published between 2015 and 2023 and were conducted in 8 countries, including 7 in Chinese, 5 in South Korea, 3 in the United Kingdom, 3 in Iran, 1 in Turkey, 1 in Saudi Arabia, 1 in the Philippines, and 1 in Indonesia. Most studies were cross-sectional (70%), with quantitative studies (86%) ranging from 106 to 1339 nursing students and qualitative studies (9%) ranging from 13 to 19 nursing students.

### Conceptual evolution

The development of academic resilience is based on resilience theory [30]. Due to researchers’ varying perspectives, several different concepts have emerged in the field of education after the introduction of the concept of resilience. Wang et al. [31]. first proposed this concept and defined it as follows: despite early traits, conditions and experiences creating an unfavorable environment, there is an increased probability of success in school. Based on previous literature, some researchers [32] have concluded that traditional academic resilience research has primarily focused on students who have suffered severe life experiences (such as changes in their living environment, illness, parental divorce, poverty, discrimination, etc.) during their early years. Despite enduring such adversity for an extended period of time, these students still managed to achieve good academic performance. The significance of academic resilience in recent risk factors (such as unsatisfactory academic performance, academic pressure, etc.) was ignored. Martin [22] et al. pointed out that academic pressure is a prevalent issue in the daily learning of any school-age student. Every student will encounter recent risk factors such as academic setbacks and pressure. Therefore, the research on academic resilience should shift from the previous minority and special groups to ordinary students. The academic resilience of this group refers to students’ ability to adjust in time after encountering academic setbacks or challenges (including poor academic performance, high levels of academic pressure, etc.) during their daily learning process, while still achieving good academic outcomes [33].

### Application of concepts

The application of academic resilience in the nursing field basically adopted the definition of academic resilience provided by Martin et al. [33]. This focuses on the role of academic resilience in the proximal influencing factors (the academic pressure perceived by nursing students in their daily learning process). In the field of nursing, these proximal influencing factors generally include adapting to unfamiliar clinical practice environments and interpersonal relationships, feelings of helplessness caused by the gap between theory and practice [34], fear of infectious diseases, and heavy workloads [35]. Jeong et al. [36] believe that academic resilience can serve as a resource for nursing students to protect their mental health from the impact of stress. It is also the ability to attain high academic achievement and sustain strong motivation and enthusiasm for university life even under stressful conditions. Li et al. [10] argue that academic resilience is specific to the academic situation, referring to students’ ability to overcome setbacks and challenges in their schoolwork. Yang et al. [37] define academic resilience as the application of resilience within the field of education. In the context of education (usually referring to schools), students are able to effectively adapt to and manage various pressures encountered during the learning process, such as setbacks and failures. This crucial ability enables them to achieve positive learning outcomes. Wang et al. [38] propose that academic resilience refers to students’ ability to recover from stressful academic situations, enabling them to effectively navigate the challenges associated with the academic achievement process. It is defined as an individual’s capacity to confidently handle, adapt to, or manage important sources of interaction in a stressful learning environment and transform negative clinical learning experiences into positive evolving outcomes. Luo et al. [39] believe that academic resilience means that nursing students can leverage personal strengths and mobilize external resources when encountering academic difficulties, ultimately leading to a positive adaptation process and successful outcomes. Shi et al. [39] define academic resilience as the ability of students to successfully cope with typical academic setbacks in school daily learning activities, reflecting their perseverance and learning capability. According to Shahidi et al. [40], academic resilience is a crucial quality of nursing students, enabling them to overcome academic pressure and adapt to the demands of both learning and clinical practice.

In summary, after conducting in-depth research on academic resilience of nursing students, researchers have presented various viewpoints. However, an exact and comprehensive definition has not yet been established.

### Definition of nursing student’s academic resilience

After conducting a thorough review and analysis of the literature, this study found that the academic resilience of nursing students is affected by a complex interaction of various factors, including internal factors of nursing students and external environmental factors. Through the analysis of the attributes, antecedents and consequences of nursing students’ academic resilience, the following definitions were obtained: when faced with academic challenges, nursing students maintain a belief in their capacity to overcome these obstacles. They proactively regulate their cognition and behavior, leverage their personal and external resources to adapt effectively to both school educational and clinical settings, ultimately achieving positive learning outcomes.

### Attributes

Attributes are a set of features or components of a concept. Clarifying the defining attributes of a concept helps to deepen the understanding of the concept, which is an important aspect of concept analysis [41]. Through literature analysis and induction, this study summarized three core attributes of nursing students’ academic resilience, including self-efficacy, self-regulation and recovery.

1. Self-efficacy.

Self-efficacy is confidence and belief [42], which refers to an individual’s belief in their ability to control outcomes, successfully complete tasks, and possess the necessary skills to accomplish those tasks [43]. It is one of the internal attributes and driving force of nursing students’ academic resilience. Improving the self-confidence of nursing students is the initial step of constructive learning, particularly when faced with academic challenges. Confidence enables students to seize learning opportunities, actively analyze and assess dilemmas they encounter, and positively impacts their ability to cope with academic pressure [38, 44].

2) Self-regulation.

Self-regulation is one of the important elements of resilience [45], which refers to the regulation of thinking, emotion, behavior and attention through deliberate or specific mechanisms. This enables individuals to effectively manage their activities over time and environmental changes [46], as well as helping them to control emotions and actions under pressure. This attribute includes cognitive regulation and behavioral regulation. Cognitive regulation: When facing academic setbacks, nursing students can positively view setbacks by cognitive reconstruction, emotional adjustment, reconstructing negative thoughts and re-attribution [47], thereby enhancing their self-efficacy to overcome difficulties. Behavioral adjustment: In the face of academic pressure or challenges, individuals have to adjust their original coping strategies, assess their own advantages, mobilize available resources within their reach, re-integrate personal resources and social resources, make the most of all available resources to overcome academic difficulties [48, 49].

3) Recovery.

Recovery is defined as the outcome of becoming well again after an illness or injury, according to the Oxford Dictionary. As one attribute of academic resilience, it refers to the outcome that nursing students rebound from adversity, and return to the right track or enter a new balance in this paper [38, 44].

### Antecedents

Antecedents refer to the events or situations that should exist prior to the development of a concept [41]. Based on Kumpfer’s psychological resilience model [49], a review of previous studies has identified two categories of antecedents for nursing students’ academic resilience: individual internal factors and external factors.

Studies have indicated that the academic resilience of nursing students is influenced by various internal factors. (1) social demographic factors: research has demonstrated that as age [40] and grade [39, 50, 51] levels increase, along with a higher educational background among nursing students [52], there is a corresponding enhancement in academic resilience. Additionally, studies have revealed that female nursing students exhibit greater academic resilience compared to male students [3, 50], while rural students tend to demonstrate higher levels of academic resilience than urban students [52]. Moreover, it has been found that non-only-child students generally display higher academic resilience when compared to only-child students [53]. (2) Spiritual factors: Studies have shown that nursing students with an optimistic attitude [44, 54], high levels of mindfulness [38, 51], strong self-confidence [44], effective self-control ability [37, 44], high learning engagement [55] and strong religious coping ability [56] are conducive to the development of academic resilience. Furthermore, nursing students who independently choose to study nursing as their major demonstrate higher academic resilience than those recommended by relatives and friends or those who are transferred into the major [52]. Additionally, nursing students with role models exhibit higher academic resilience than those without role models [3]. (3) Emotional factors: Research has indicated that higher levels of emotional intelligence [44, 54] is linked to increased academic resilience among nursing students. In addition, when faced with academic setbacks, nursing students may employ different coping styles [36], which can also impact their academic resilience. (4) Cognitive factors: Literature has shown that nursing students with high levels of academic performance [3, 36, 40, 57], scholarship [52], self-directed learning ability [54] and skill level [38] also have high academic resilience. (5) Physical factors: Nursing students with good subjective health status demonstrate higher academic resilience than those with poor subjective health status [54]. (6) Behavioral factors: Nursing students’ interpersonal communication ability [57] are also influential factors in their academic resilience.

The level of nursing students’ academic resilience is not only affected by internal factors of individuals, but also restricted by external factors. (1) school factors: Studies have indicated that high pressure in school life [54] and low professional satisfaction [3, 36, 54] are hindering factors for nursing students’ academic resilience. Moreover, a positive clinical work atmosphere [38], strong interpersonal relationships at school [3], encouragement and support from teachers [38, 44], as well as support from patients and their families [38] and recognition [38] during clinical practicum were promoting factors of academic resilience. (2) Family factors: Literature has shown that high levels of family relationship satisfaction [3], effective family communication mode [57] and strong family support [38] contribute to the improvement of academic resilience. (3) Peer group factors: Peer support [38, 58] and good peer’s interpersonal relationship [3] can also promote the enhancement of nursing students’ academic resilience.

### Consequences

Consequences refer to the events or situations caused by the concept [41]. Studies [21, 38, 39, 52, 55, 59] have indicated that academic resilience plays a significant role in enhancing nursing students’ adaptability during the school period, career maturity, adversity quotient level, probability of academic success and a sense of belonging to school. Additionally, it has been found to reduce the degree of psychological distress experienced by nursing students. “Career maturity” refers to the extent to which individuals are able to accomplish tasks that are coordinated with their stage of career development [52]. “Adversity quotient level” refers to an individual’s ability to cope with setbacks and adversity [39].

### Related concepts

Related concepts refer to words that share commonalities with concepts but do not possess identical characteristics. Academic buoyancy refers to a student’s capacity to successfully cope with the academic setbacks and challenges typical of school life. Unlike academic resilience, academic buoyancy is more closely related to how a student handles the everyday stressors commonly experienced by students, such as tight deadlines, challenging assignments, exam stress, and unexpected or persistent low grades. However, resilience plays a role when students suffer from long-term poor performance, overwhelming anxiety that they cannot bear, truancy, and significant setbacks due to dissatisfaction [60]. Academic hardiness is defined as the ability to prepare oneself to confront academic problems. It comprises three components: control (the ability to manage different life situations), commitment (willingness to engage) and challenge (the ability to understand that changes in life are normal), emphasizing individuals’ endurance in the face of difficulties [61, 62].

### Model case

The concept analysis of model case is beneficial to better understand and identify the connotation of concepts [41]. Although Rodgers does not suggest the creation of a model case, in order to better understand the concept, we used Walker and Avant’s [29] method to construct a model case as follows.

Lucy, 22 years old, is a member of a family consisting of four members (father, mother and brother), with very harmonious family relations. As a junior student, Lucy has a passion for medicine and has taken the initiative to study nursing at university in pursuit of a bachelor’s degree. After three years of theoretical study, she entered a large hospital to begin clinical practice and complete the final stage of her undergraduate education. Lucy’s first internship took place in the emergency ward of a hospital. Faced with an unfamiliar clinical environment, heavy medical workloads, anxious patients and their families, as well as sudden situations (such as the rescue of patients and unexpected deaths, etc.), Lucy experienced significant pressure. Due to the gap between theoretical knowledge and clinical practice and inexperienced nursing skills, Lucy was afraid of providing nursing interventions for patients and making mistakes. Every day when she went to the hospital for her internship, Lucy felt extremely anxious. During conversations with her family members, Lucy confided in them about her troubles. Her family listened patiently to Lucy’s difficulties and offered positive affirmation and encouragement. They believed that Lucy would overcome the current difficulties and become a qualified nurse. With the encouragement of her family, Lucy recalled her initial decision to pursue a career in nursing voluntarily, aspiring to be someone who could alleviate patients’ suffering like Florence Nightingale. She then adjusted her mindset and earnestly contemplated how to confront and surmount the present difficulties with a positive outlook. She firmly believed that she could triumph over the current predicament through her diligent efforts. Lucy took the initiative to contact the senior students and inquire about their experiences in adapting to the clinical environment during their internship. Drawing on their experience, she utilized her solid theoretical knowledge from the initial three years of study for understanding new knowledge in clinical work. Throughout her internship, Lucy carefully observed the operations of her mentor, diligently recorded any challenges encountered, sought clarification when needed, and dedicated herself to repeated practice. The mentor also affirmed Lucy’s efforts and gave timely feedback to her operation, which significantly enhanced Lucy’s nursing skills and bolstered her confidence. In order to maintain a healthy physical state for clinical work, Lucy conscientiously balanced her study and rest time. Additionally, she incorporated mindfulness exercises into her routine to relax both body and mind while improving focus and reducing distress related to professional challenges. Through her efforts, Lucy earned recognition from patients and their families while experiencing a profound sense of accomplishment when performing patient care procedures. Finally, she successfully completed her internship tasks and obtained a bachelor’s degree in nursing.

Lucy’s case exemplifies the three attributes of nursing students’ academic resilience, namely self-efficacy, self-regulation and recovery. Upon entering the clinical practice stage, Lucy experienced significant pressure but maintained a belief in her ability to overcome challenges through diligent effort. She actively analyzed her current station and seized learning opportunities, reflecting the first attribute of academic resilience, self-efficacy. Through cognitive reconstruction and emotional adjustment, Lucy approached her difficulties with a positive attitude and effectively utilized her strengths to navigate the challenges she faced. For instance, she has established a solid foundation for theoretical learning, confided her difficulties with her family, sought assistance from her seniors and mentors, maintained good health, utilizing available internal and external resources around her. By regulating one’s own cognition and behavior to overcome academic challenges, demonstrating the second attribute of academic resilience, self-regulation. By actively analyzing the dilemma she faced, adjusting her own thoughts and behaviors, integrating internal and external resources, Lucy successfully rebounded from adversity. She adapted to the clinical pressure environment and ultimately completed the internship task. This reflects the third attribute recovery of academic resilience. The stress induced by clinical practice, Lucy’s optimistic attitude, high levels of mindfulness, independent choice of nursing major, role model, high levels of emotional intelligence, positive coping style, good academic achievement, healthy physical state, high self-directed learning ability, self-control ability, interpersonal communication ability, skill level, support from mentors, family members, patients and their family members, peers all contribute to the enhancement of Lucy’s academic resilience. The improvement of Lucy’s adaptability, career maturity, adversity quotient level and the reduction of her psychological distress during her clinical internship are the consequences of her academic resilience.

### Empirical referents

In order to gain a better understanding of the concept, we used Walker and Avant’s [29] method to provide empirical referents. Currently, there are two primary tools for evaluating nursing students’ academic resilience. One is the Nursing Student Academic Resilience Inventory (NSARI) developed by Iranian scholar Ali-Abadi [27], which comprises a total of 24 items. The six dimensions include optimism (5 items), communication (4 items), self-esteem/evaluation (4 items), self-awareness (3 items), trustworthiness (4 items) and self-regulation (4 items). The scale employed a 5-point Likert scale ranging from completely agree (5 points) to completely disagree (1 point), with scores requiring conversion into standardized scores. Higher scores indicate higher levels of academic resilience. The intraclass correlation coefficient of the scale was 0.903, and the Cronbach’ α coefficient of the scale was 0.66–0.78, indicating good reliability and validity. This instrument was developed with undergraduate nursing students as the research subjects and specifically used to measure the academic resilience of nursing students. It has been applied to undergraduate nursing students in Iran [40]. Additionally, Turkish scholar Enes Ucan [63] et al. conducted cross-cultural adaptation of the tool.

The other is Academic Resilience Inventory for Nursing Students (ARINS) developed by Li Chengjie [10], a scholar from Taiwan, China. The scale contains a total of 15 items, including three dimensions: cognitive maturity (5 items), emotional regulation (3 items) and help-seeking resources (7 items). The 5-point Likert scale ranging from “very inconsistent” (1 point) to “very consistent” (5 points) was employed, with higher scores indicating higher levels of academic resilience. The Cronbach’s α coefficient of the total scale was 0.929. The Cronbach’s α coefficients of the three dimensions of cognitive maturity, emotional regulation and help-seeking resources were 0.926, 0.801 and 0.855, respectively. The theoretical model exhibited a good fit with the observation data. The total scale demonstrated excellent reliability and validity. Originally designed to measure the academic resilience of nursing students holding junior college degrees in Taiwan. Additionally, it has been applied in undergraduate nursing students in Chinese mainland [37, 55] while showing good reliability and validity. However, further investigation is required to determine these tools’ applicability among nursing students at different educational levels.

## Disscussion

### The conceptual model of nursing students’ academic resilience

**Fig. 2:**
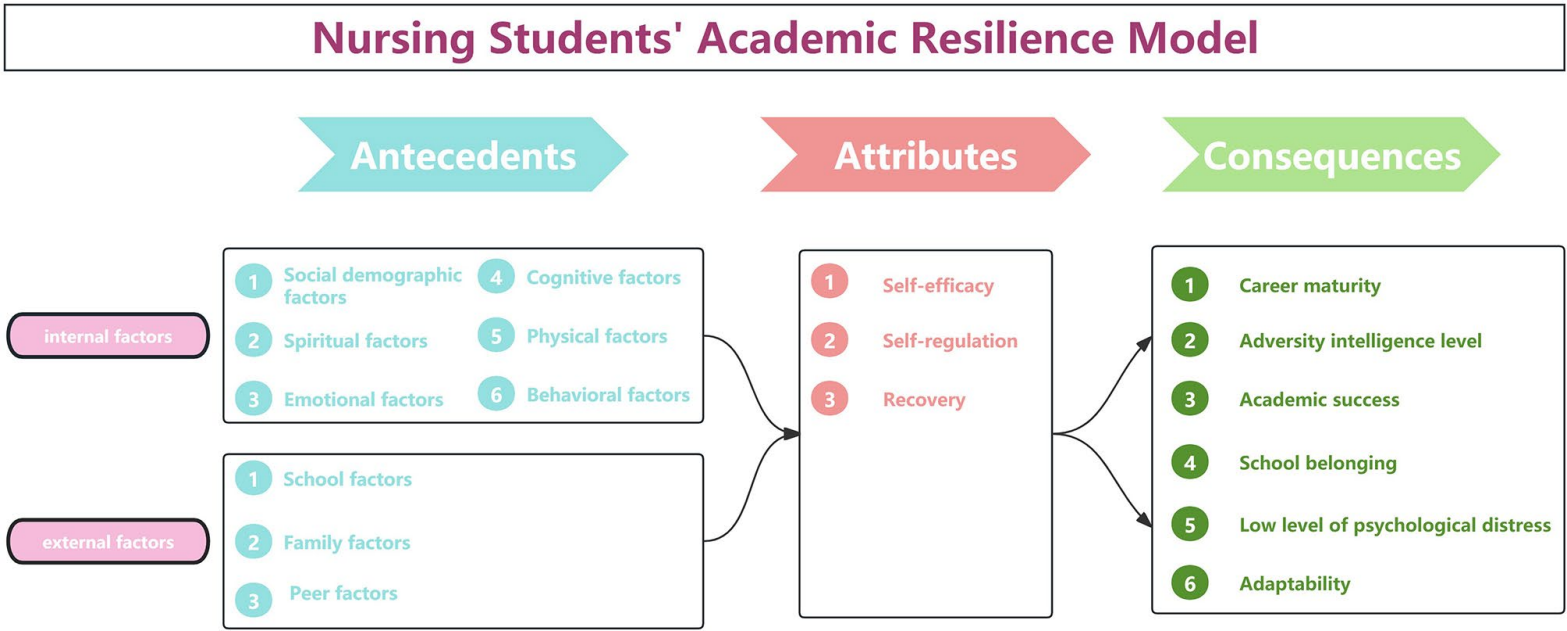
Model of nursing student’s academic resilience

Through a comprehensive analysis and synthesis of literature, this study has developed a conceptual model of nursing students’ academic resilience, as shown in Fig. 2. In essence, the academic resilience of nursing students is shaped by the interplay between internal and external factors when they encounter setbacks or challenges in their academic pursuits. This interaction ultimately fosters the development of academic resilience among nursing students and yields positive outcomes. Nursing students with high levels of academic resilience are able to view academic setbacks positively, enhance their problem-solving skills, and effectively navigate through academic difficulties, thereby successfully completing their studies and go to the future nursing position [42].

### The significance of the role of nurses for the nursing students’ academic resilience

The definition and attributes of nursing students’ academic resilience proposed in this study emphasize the characteristics of nurses’ working environment and learning environment of nursing students. Nurses play a crucial role as providers of healthcare services, taking on important medical tasks with increasingly complex professional responsibilities. They possess multifaceted roles as healthcare providers, strategists, administrators, educators, and coordinators [64, 65]. This necessitates not only a solid foundation of professional knowledge and skills but also profound critical thinking and judgment abilities, astute observational and analytical capabilities, decisive decision-making prowess, and exceptional interpersonal communication aptitude [66]. These qualities enable them to effectively manage busy and high-pressure clinical tasks within an increasingly tense doctor-patient relationship, ultimately providing satisfactory medical services to patients and their families. As the reserve army of the nursing team, nursing students are expected to quickly acquire these knowledge and abilities during their theoretical learning and clinical practice, thereby imposing significant academic pressure on them [67]. Simultaneously, it is these qualities that distinguish nursing from other specialties and healthcare disciplines. It is hoped that the results of this concept analysis will inspire further research into effective measures aimed at enhancing the academic resilience of nursing students, in order to improve their ability to cope with academic setbacks and increase retention rates of nurses.

### Implications and recommendations

Currently, there are relatively few studies on the academic resilience of nursing students, and there are significant discrepancies in the definition of conceptual attributes among nursing students’ academic resilience in the existing literature. This study addresses this gap by providing a comprehensive conceptualization of academic resilience of nursing students by combing the related concepts, attributes, antecedence and consequences of nursing students’ academic resilience. The findings offer a theoretical foundation for future research.

Academic resilience plays a crucial role in regulating individuals’ pressure levels, enhancing their problem-solving abilities, and supporting nursing students in successfully completing their academic studies and transitioning into future nursing positions [42]. This study provides a conceptual framework for nursing schools and hospitals to design academic resilience training curricula and programs for nursing students. It is essential to thoroughly investigate these relationships based on the nursing context through appropriate measures. This process can clearly define attributes, antecedence, and consequences of nursing students’ academic resilience. Nursing educators can begin by focusing on the antecedence and attributes of nursing students’ academic resilience. This can be achieved through targeted training programs or specialized courses aimed at improving emotional intelligence, interpersonal communication skills, self-directed learning capabilities, self-control, self-regulation, and self-efficacy among nursing students. In addition, nursing educators can also consider external environmental factors. This includes creating a conducive learning and working atmosphere for nursing students, providing training for professional teachers and clinical instructors, setting a professional example for nursing students, offering timely guidance, feedback and encouragement to support students’ learning. Moreover, it’s important to guide nursing students to think positively, helping them build self-confidence, and enhance their academic confidence. Additionally, peer support programs can be designed. Existing research [68] suggests that interaction and practice among nursing students with similar knowledge, skills, and experience can promote the effective learning. The experience of other students in internship and studies can help build confidence and resilience in academic research while reducing concerns about academic issues and clinical practice. It is helpful for nursing students to improve their self-esteem and confidence in academic success which can enhance their resilience.

This study has discovered that academic resilience of nursing students is associated with several key outcomes, including academic success, career maturity, adversity quotient and school sense of belonging. Additionally, it has been found to have a positive impact on reducing psychological distress among nursing students. As a result, nursing educators can consider targeting academic resilience as an intervention strategy in order to indirectly improve these outcomes for students.

### Limitations

Due to language limitations, only English and Chinese literature were included in this study. Although the relevant literature was searched as comprehensively and scientifically as possible, grey literature sources were not included. In addition, due to limitations of some database resources, 14% of the literature was not obtained in full text. Nevertheless, this study explored the concept, attributes, antecedents and consequences of nursing students’ academic resilience through the obtained studies and achieved the research purpose.

## Conclusion

This study applied Rodgers’ evolutionary concept analysis method to clarify the conceptual attributes, antecedents, consequences and related concepts of academic resilience among nursing students, and combined with model case to help understand the concept. The clear definition of nursing students’ academic resilience is beneficial for both nursing educators and nursing students themselves. It allows them more accurately assess the level of academic resilience and explore effective intervention measures to improve the level of academic resilience and increase the probability of academic success of nursing students.

## Electronic supplementary material

Below is the link to the electronic supplementary material.

Supplementary Material 1

## Data Availability

All data generated or analyzed during this study is included in this published article.
