# Supplementary material for: Academic resilience in nursing students: a concept analysis
Source: BMC Nurs. 2024 Jul 9;23:466. doi: 10.1186/s12912-024-02133-2 (PMC11232226; doi:10.1186/s12912-024-02133-2)
Supplement: Supplementary file 1 — Supplementary Material 1 [file 12912_2024_2133_MOESM1_ESM.docx]

**Search Strategy**

| Datebase | Search Strategy |
| --- | --- |
| PubMed | ((nursing[Title/Abstract]) AND (student*[Title/Abstract])) AND (academic resilienc*[Title/Abstract]) |
| Web of Science | ((TI=(nursing) AND TI=(student*)) AND TI=(academic resilienc*)) OR ((AB=(nursing) AND AB=(student*)) AND AB=(academic resilienc*)) |
| Scopus | ( TITLE-ABS-KEY ( nursing ) AND TITLE-ABS-KEY ( student* ) AND TITLE-ABS-KEY ( academic AND resilienc* ) ) |
| CINHAL | (TI nursing AND TI student* AND TI academic resilienc* ) OR (AB nursing AND AB student* AND AB academic resilienc*) |
| PSYCINFO | (TI nursing AND TI student* AND TI academic resilienc* ) OR (AB nursing AND AB student* AND AB academic resilienc*) |
| Science Direct | Title, abstract, keywords: nursing and students and (academic resilience) |
| ProQuest | ([title(nursing) AND title(student*) AND title(academic resilienc*)](https://www.proquest.com/recentsearches.recentsearchtabview.recentsearchesgridview.scrolledrecentsearchlist.checkdbssearchlink:rerunsearch/67939D4C9E654DD8PQ/None/$N?t:ac=RecentSearches)) OR ([abstract(nursing) AND abstract(student*) AND abstract(academic resilienc*)](https://www.proquest.com/recentsearches.recentsearchtabview.recentsearchesgridview.scrolledrecentsearchlist.checkdbssearchlink:rerunsearch/A75B9C98246C4A95PQ/None/$N?t:ac=RecentSearches)) |
| Embase | nursing:ab,ti AND student*:ab,ti AND 'academic resilienc*':ab,ti |
| CNKI | SU=(academic resilience) AND SU=(nursing) AND SU=(student) |
| Wanfang Database | SU=(academic resilience) AND SU=(nursing) AND SU=(student) |
| VIP database | (T=(academic resilience) AND T=(nursing) AND T=(student)) OR (R=(academic resilience) AND R=(nursing) AND R=(student)) |
